# Administration of probiotic lactic acid bacteria to modulate fecal microbiome in feedlot cattle

**DOI:** 10.1038/s41598-022-16786-z

**Published:** 2022-07-28

**Authors:** Flavia Ivana Mansilla, Cecilia Aristimuño Ficoseco, María Hortencia Miranda, Edoardo Puglisi, María Elena Fatima Nader-Macías, Graciela Margarita Vignolo, Cecilia Alejandra Fontana

**Affiliations:** 1 Centro de Referencia Para Lactobacilos (CERELA), CONICET, Tucumán, Argentina; 2 Instituto Nacional de Tecnología Agropecuaria INTA EEA-Famaillá, Tucumán, Argentina; 3 Present Address: Dipartimento di Scienze e Tecnologie Alimentari per una Filiera Agro-alimentare Sostenibile (DISTAS), Università Cattolica del Sacro Cuore, Cremona-Piacenza, Italy

**Keywords:** Animal behaviour, Applied microbiology, Microbial communities

## Abstract

Modulation of animal gut microbiota is a prominent function of probiotics to improve the health and performance of livestock. In this study, a large-scale survey to evaluate the effect of lactic acid bacteria probiotics on shaping the fecal bacterial community structure of feedlot cattle during three experimental periods of the fattening cycle (163 days) was performed. A commercial feedlot located in northwestern Argentina was enrolled with cattle fed mixed rations (forage and increasing grain diet) and a convenience-experimental design was conducted. A pen (n = 21 animals) was assigned to each experimental group that received probiotics during three different periods. Groups of n = 7 animals were sampled at 40, 104 and 163 days and these samples were then pooled to one, thus giving a total of 34 samples that were subjected to high-throughput sequencing. The microbial diversity of fecal samples was significantly affected (*p* < 0.05) by the administration period compared with probiotic group supplementation. Even though, the three experimental periods of probiotic administration induced changes in the relative abundance of the most representative bacterial communities, the fecal microbiome of samples was dominated by the Firmicutes (72–98%) and Actinobacteria (0.8–27%) phyla, while a lower abundance of Bacteroidetes (0.08–4.2%) was present. Probiotics were able to modulate the fecal microbiota with a convergence of *Clostridiaceae*, *Lachnospiraceae*, *Ruminococcaceae* and *Bifidobacteriaceae* associated with health and growth benefits as core microbiome members. Metabolic functional prediction comparing three experimental administration periods (40, 104 and 163 days) showed an enrichment of metabolic pathways related to complex plant-derived polysaccharide digestion as well as amino acids and derivatives during the first 40 days of probiotic supplementation. Genomic-based knowledge on the benefits of autochthonous probiotics on cattle gastrointestinal tract (GIT) microbiota composition and functions will contribute to their selection as antibiotic alternatives for commercial feedlot.

## Background

Probiotic preparations have shown promising results in a variety of animal production areas. During the past 30 years, the marketing of direct-fed microbes has significantly increased in all sectors of livestock agriculture. A major reason for this expansion was directed toward replacement of low-dose antibiotics as a common practice for improving overall animal health with benefits on increased feed efficiency, body weight and performance^1–3^. However, this production practice has received a great deal of scrutiny owing to the increased attention to the use of medically important antimicrobials in livestock husbandry. An emerging concern regarding antibiotic use in meat animals is the selection for bacterial communities that are more resistant to antibiotics, thus posing a threat to consumer health and a negative effect on the environment^4^. The growing concern over the transmission of antimicrobial resistance to humans through the food chain or environmental routes has resulted in the need to decrease their use and search for alternatives. Since the ban of in-feed antibiotics by European legislation in 2006, the decline in their use has enabled the reduction of resistant intestinal bacteria prevalence^5^. Therefore, antibiotic use as growth enhancers in livestock diets faces widespread bans across many countries^6^.

Among strategies for antibiotic replacement, the use of feed additives favorably affects animal performance and welfare, particularly through gut microbiota modulation, generated a great deal of research. A balanced gut microbiota constitutes an efficient barrier against pathogen colonization, produces beneficial metabolic substrates and stimulates the immune system in a noninflammatory manner^7,8^. In this context, probiotics, defined as microbial feed supplements that beneficially affect the host animal, have generated many expectations. They are able to inhibit pathogenic microorganisms by producing antimicrobial compounds such as bacteriocins and organic acids and regulate the gastrointestinal (GI) environment by adhesion to intestinal mucosa, preventing attachment of pathogens and competing for nutrients to decrease the load of pathogenic bacteria^8^. Furthermore, other positive effects of probiotics as an alternative to feed additives include improved dry matter intake and feed conversion efficiency, increased nutrient utilization and production performance, and reduced methane production, thereby minimizing energy loss, overall growth, health promotion and meat and milk production in ruminants^3,9^.

Specifically, probiotics for ruminants include direct-fed microbes such as yeasts (*Saccharomyces cerevisiae*) and bacterial species including *Bacillus*, *Bifidobacterium*, *Propionibacterium* and lactic acid bacteria (LAB). *Lactobacillus*, *Pediococcus* and *Enterococcus* species are the LAB probiotics most applied in feedlot cattle^10–14^ and show a positive influence on various digestion processes, especially on cellulolytic degradation of plant material and the synthesis of microbial proteins^13^. In addition, as immunobiotics, they are also gaining interest in animals of economic importance for reducing the incidence of infections and increasing productive performance^8,15,16^. Indeed, probiotic strains have been reported to regulate the innate immune response, inducing the upregulation of TLR negative regulators while reducing the expression of proinflammatory cytokines and chemokines in bovine intestinal epithelial cells^17^. Probiotic administration to bovine cattle has also received increased interest as a method to reduce foodborne pathogenic bacteria in cattle feces; in particular, the presence of enterohemorrhagic *Escherichia coli* O157:H7 (EHEC) in the gastrointestinal tract has been linked to disease outbreaks due to the consumption of contaminated beef/dairy foods and water^18,19^. Thus, research efforts have been directed to reduce fecal shedding of EHEC by preharvest interventions such as the use of probiotic administration^20,21^.

Because of the enormous influence of the cattle fecal bacterial community on the beef and dairy industry, public health, animal diseases and productivity, a great deal of research has been directed to characterize the effects of animal age, feeding diets and operations practices, as well as antibiotic treatments on the structure of cattle fecal microbiota^22–26^; however, the effect of LAB on cattle feces has been scarcely investigated. To obtain access to the complete bacterial repertoire within bovine fecal samples, advances in high-throughput sequencing (HTS) techniques now enable the mapping of microbial communities in the gastrointestinal tract^27–29^. Furthermore, HTS was able to redefine the rumen microbiome and its relationship with nutrition and metabolism, which would be the key to enhancing animal production efficiency^30^. In previous works, the identification of LAB from a feedlot environment^31^ and their characterization as probiotics^32^ were performed. Thus, the aim of this study was to investigate by means of HTS, the effect of probiotic administration on the fecal microbiota structure and function of feedlot cattle adapted to a high-energy diet during three periods of the intensive fattening cycle.

## Results

### Effect of administered probiotics on the bacterial community structure of fecal samples

**Figure 1:**
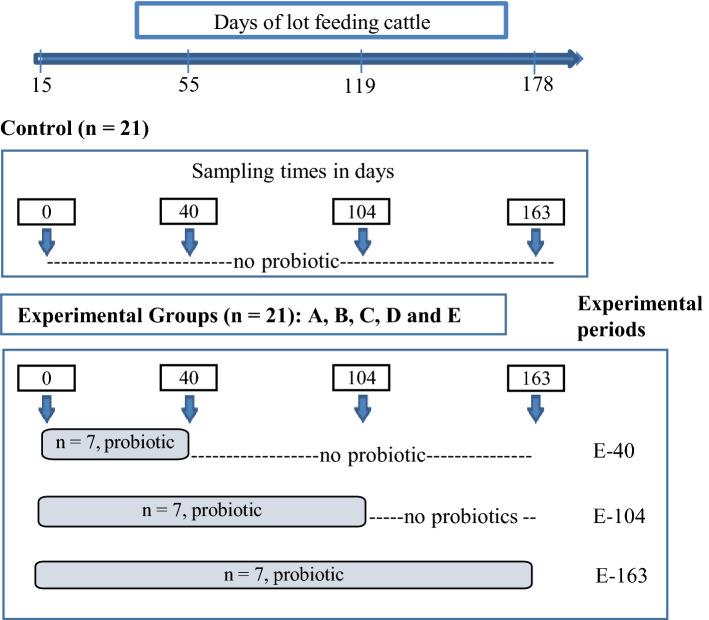
Experimental design: probiotic experimental groups: A: *Lactobacillus acidophilus* CRL 2074; B: *Limosilactobacillus fermentum* CRL 2085; C: *Limosilactobacillus mucosae* CRL 2069; D: CRL 2085 + CRL 2069; E: CRL 2074 + CRL 2085 + CRL 2069; Control group (n = 21) included in a separate pen. Sampling time in days: 0 (T0), 40 (T1), 104 (T2) and 163 (T3). Experimetal periods of probiotics administration E-40, E-104 and E163. Sampling scheme: animals (n = 7) received probiotics up to 40 days and then removed (to separate pen) experimental period E-40; animals (n = 7) received probiotics up to 104 days and then removed, E-104 and, animals (n = 7) administered with probiotics for 163 days E-163. This scheme was replicated for the A, B, C, D and E experimental groups.

**Figure 2:**
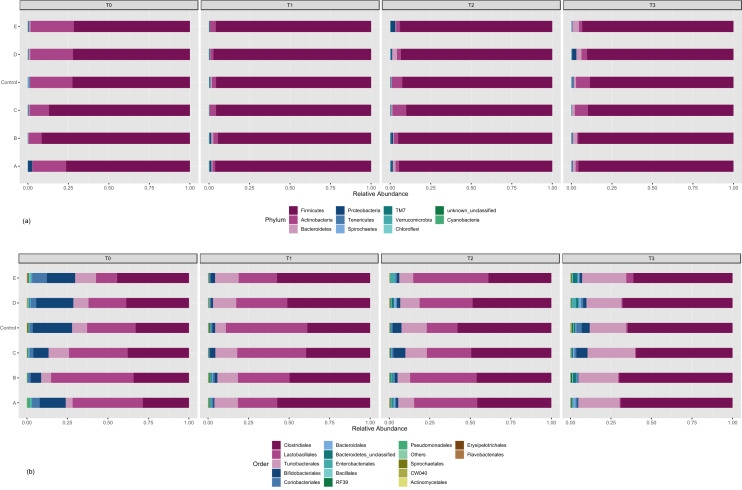
Phylum- (**a**) and order-level (**b**) distribution of the microbiome in probiotics (A: *Lactobacillus acidophilus* CRL 2074*;* B*: Limosilactobacillus fermentum* CRL 2085*;* C: *Limosilactobacillus mucosae* CRL 2069*;* D: CRL 2085 + CRL 2069*;* E**:** CRL 2074 + CRL 2085 + CRL 2069-treated and untreated (control) fecal samples of feedlot cattle at different sampling times (T0: 0 day; T1: 40 days; T2: 104 days; T3: 163 days). Each color represents a phylum and an order.

Metagenomics from 16S rDNA was carried out on fecal samples to study the effect of probiotic strains on the structural composition of the gut microbiota. A convenience-experimental design and sampling scheme used in this study is shown in Fig. 1 (see also Material and Methods section). A total of 1.649.224 high-quality sequences were analyzed, and the average count per sample was 68.717. The achieved coverage of the total bacterial diversity was over 99.9% (Fig. 1). The overall number of OTUs detected by analysis was 16.381 based on 97% nucleotide sequence identity between reads. First, we explored fecal microbiota modulation for each experimental probiotic group (A, B, C, D, E and control) during 163 days of continuous administration by analyzing four different sampling times. A graphical representation of the microbial transition during 163 days of different probiotic administrations is presented in Fig. 2. Taxonomic assignment at the phylum level showed Firmicutes (71–97%), which was predominantly present in all samples throughout the whole experiment (Fig. 2a). Actinobacteria (1–27%), the second most abundant phylum, exhibited differences in the relative abundance depending on the administered probiotic, although it was higher at T0, while Bacteroidetes (0.3–4%) showed an increase over time. Proteobacteria, Tenericutes, Spirochaetes, TM7, Cyanobacteria, Chloroflexi and Verrucomicrobia were also identified, and their relative abundance varied with the administered probiotic group. The composition of the bacterial community at the order level (Fig. 2b) displayed a similar distribution pattern as that of the phyla. Twenty-seven bacterial orders were identified, but only 16 accounted for ≥ 1% of sequences overall. Control fecal samples on T0 accounted for the highest diversity among all fecal samples. Lactobacillales, Bifidobacteriales and Coriobacteriales were the most represented, and a general reduction in these taxa occurred at T3 (163 days). Notably, an enrichment in the abundance of Turibacterales was produced and together with Clostridiales, accounted for the greatest prevalence for control and treated samples at T3 (163 days); the highest abundance was found in the presence of probiotic C-163 (L. mucosae CRL2069) at T3. Bacteroidales and Enterobacteriales relative abundances correlated with those at the phylum level.

**Figure 3:**
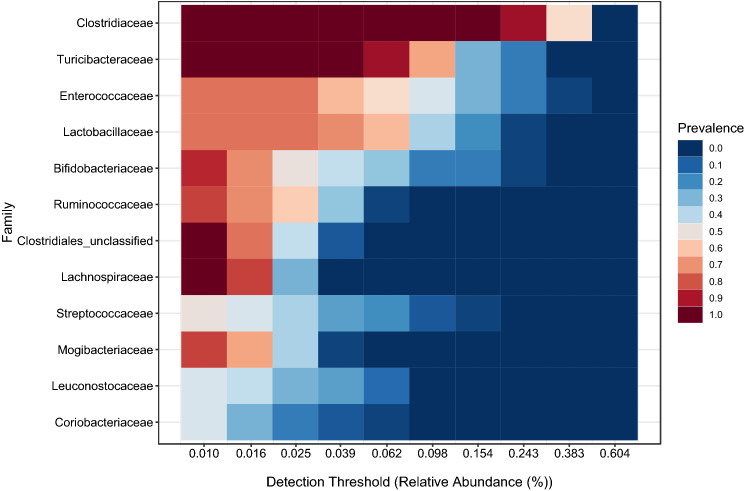
Core microbiome at the family level of feedlot cattle fecal samples displayed by a heatmap generated by pipeline Microbiome Analyst (http://microbiomeanalyst.ca/faces/home.xhtml). Color shading indicates the prevalence of each bacterial family among samples for each abundance threshold. As we increased the detection threshold, the prevalence decreased.

Although approximately 48 families (including taxa with sequences > 10) were identified in the analyzed samples (Fig. S2), twelve of them, *Clostridiaceae*, *Turicibacteriaceae*, *Enterococcaceae*, *Lactobacillaceae*, *Ruminococcaceae*, unclassified Clostridiales, *Lachnospiraceae*, *Streptococcaceae, Leuconostoccaceae*, *Bifidobacteriaceae Coriobacteriaceae* and *Mogibaceriaceae*, constituted the core microbiota of the analyzed feedlot cattle fecal samples (Fig. 3).

**Figure 4:**
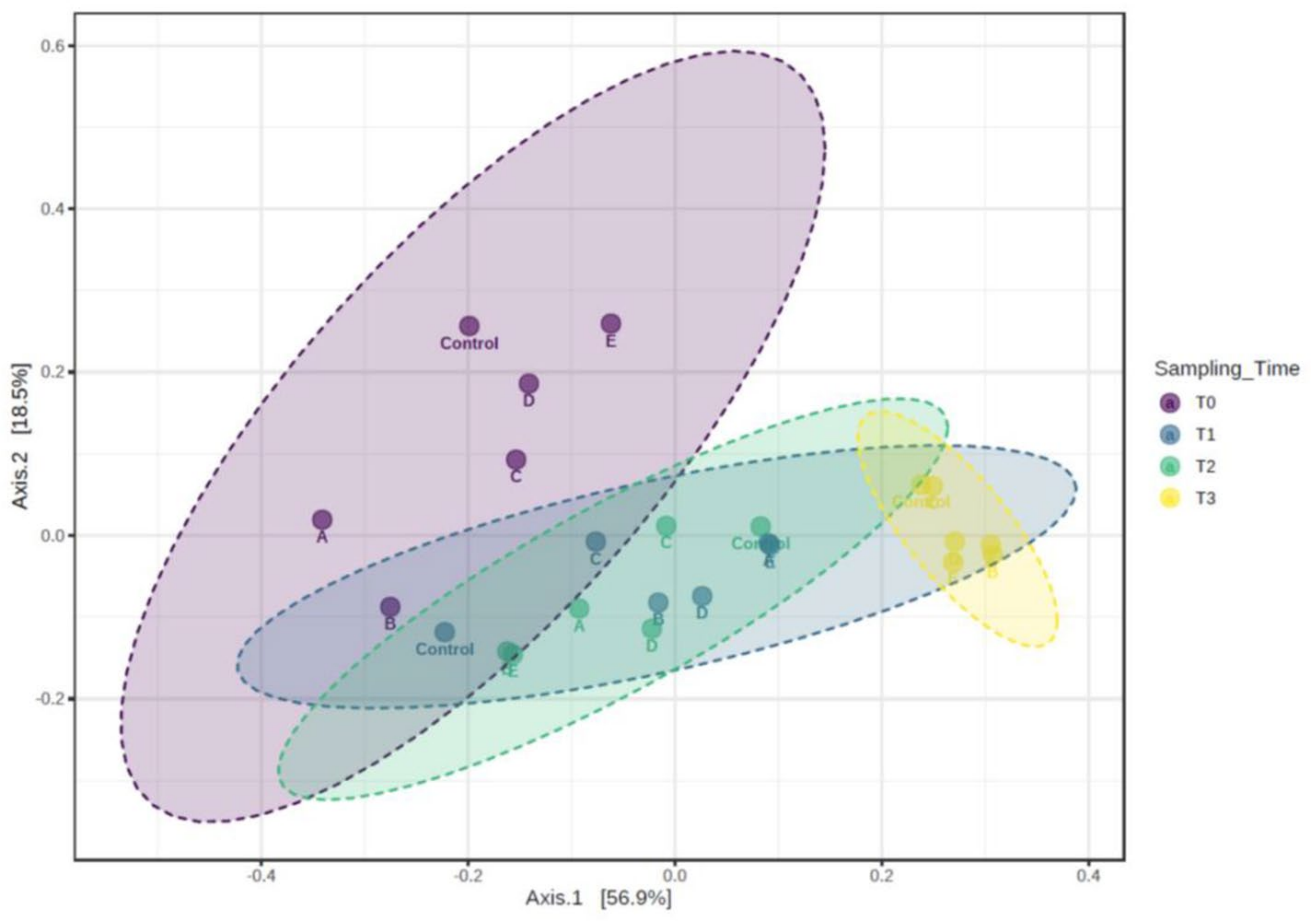
Principal coordinate analysis (PCoA) based on Bray Curtis ß-diversity. The plot illustrates the distances between bacterial communities in all individual fecal samples (A: *Lactobacillus acidophilus* CRL 2074*;* B*: Limosilactobacillus fermentum* CRL 2085*;* C: *Limosilactobacillus mucosae* CRL 2069*;* D: CRL 2085 + CRL 2069*;* E: CRL 2074 + CRL 2085 + CRL 2069 treated and untreated (control) at different sampling times (T0, T1, T2 and T3).

Moreover, to better assess the relationships among microbial communities at the family level, PCoA based on the β-diversity/Bray–Curtis and PERMANOVA analyses was performed. The plot in Fig. 4 illustrates the distances between bacterial communities in all individual samples (contribution rate of 56.9% for the first principal component and 18.5% for the second component). Significant differences (*p* < 0.001) were observed between the different sampling times, highlighting the dynamic changes in the microbial community relative abundances occurring during 163 days of probiotic administration; a clear cluster tendency was observed regardless of the probiotic groups.

Alpha diversity analysis also revealed significant differences (*p* < 0.05) for the species richness (Chao1) and Shannon indices (Fig. S3, considering the different sampling times; a decrease in the richness (Chao1) and diversity (Shannon) values during the feeding periods of the animals in the lot were observed at T3. In all probiotic-treated groups (A, B, C, D and E), the highest Chao1 values were evidenced at T2 (104 days).

### Effect of experimental administration periods on the bacterial community structure of fecal samples

**Figure 5:**
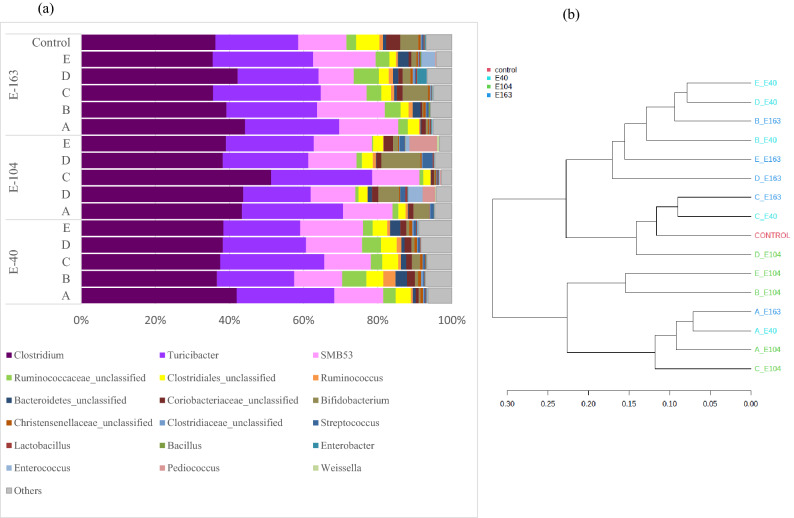
Genus-level distribution (**a**) of the microbiome in probiotic-treated (A: *Lactobacillus acidophilus* CRL 2074*;* B*: Limosilactobacillus fermentum* CRL 2085*;* C: *Limosilactobacillus mucosae* CRL 2069*;* D: CRL 2085 + CRL 2069*;* E: CRL 2074 + CRL 2085 + CRL 2069) and untreated (control) fecal samples of feedlot cattle analyzed at 163 days (T3) after different administration periods. Each color represents a genus. (**b**) Dendrogram representing hierarchical clustering distances based on Bray–Curtis dissimilarity indices calculated at the genus level. For each probiotic group and control sample, T3 was considered.

To understand how probiotic administration during the three experimental periods, E-40, E-104 and E-163 (Fig. 1 and Fig. S1) differentially modulated the fecal microbiota, each probiotic group was analyzed at T3. The alpha diversity analysis evidenced significant differences (*p* < 0.05) in the richness, abundance (number of different species) and diversity among samples (Fig. S4. For all probiotic groups, richness evidenced by the Chao1 index was higher for the E-40 and E-163 experimental periods than for the E-104 experimental period, while the highest Shannon values (diversity) were observed during E-40, and the lowest value was observed for group E-104. The three experimental periods of probiotic administration induced changes in the relative abundance of the most representative bacterial communities. The analysis of taxa at the genus level showed specific profiles for each sample; however, a tendency to cluster was observed through a Bray–Curtis dissimilarity analysis (Fig. 5a, b). Seventy-nine different genera were identified, but only 18 accounted for ≥ 2% of sequences overall (Fig. 5a), with *Clostridium*, *Turicibacter* and the candidate *SMB53* genus (within the *Clostridiaceae* family) as the most abundant in all samples at T3 (163 days). A dendrogram (Fig. 5b) revealed the occurrence of two main clusters containing two smaller clusters; cluster I included mostly E-104 experimental time samples, while cluster II contained all samples at experimental times E-163 and E-40. From this result, it can be inferred that samples from different periods of probiotic group A *L. acidophilus* CRL2074) exhibited the same community abundance at the genus level (cluster 1), whereas similar abundances were observed for the other probiotic groups at E-40 and E-163 (cluster II).

**Figure 6:**
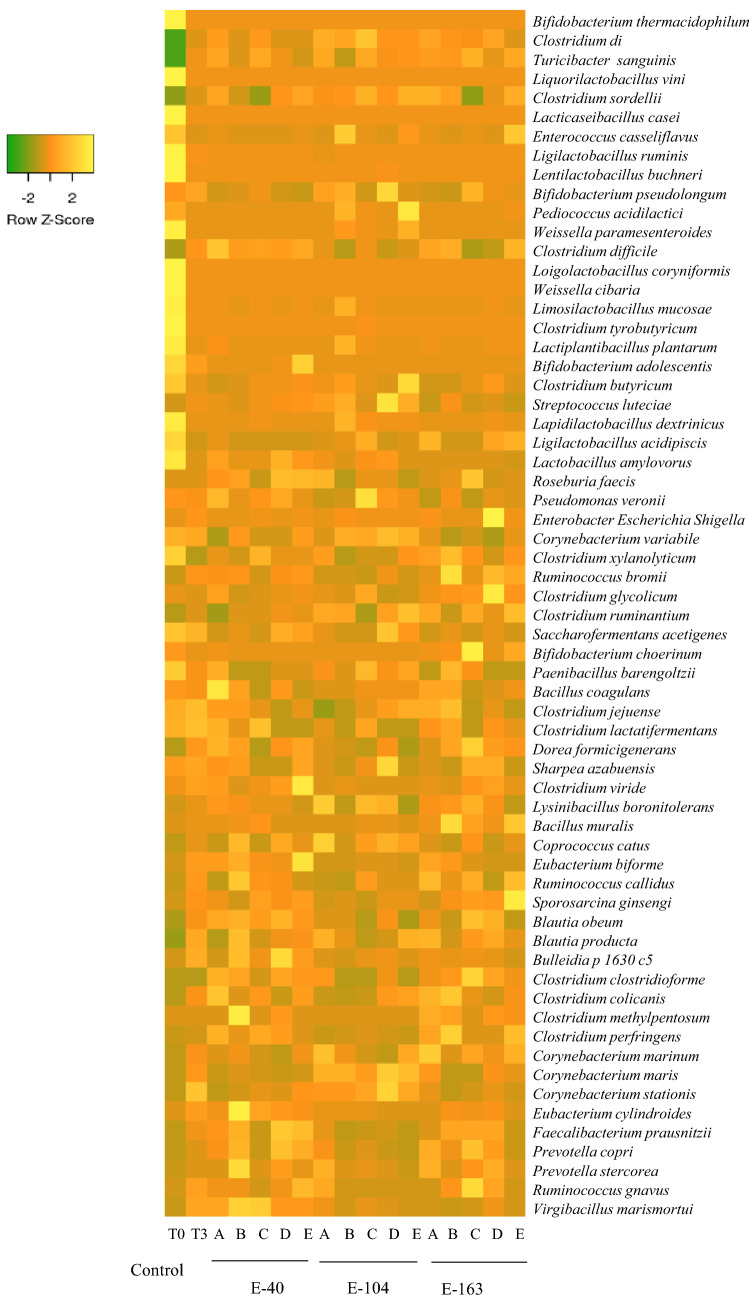
Heatmap showing the species profile in probiotic-treated (A: *Lactobacillus acidophilus* CRL 2074*;* B: *Limosilactobacillus fermentum* CRL 2085; C: Limosilactobacillus mucosae CRL 2069*;* D: CRL 2085 + CRL 2069; E: CRL 2074 + CRL 2085 + CRL 2069) and untreated (control) feedlot fecal samples at different experimental administration periods (E-40, E-104 and E-163). For each probiotic group, samples at T3 were considered, while for controls T0 and T3, samples were analyzed. The figure was generated using the heatmap web server http://www.heatmapper.ca/expression/.

More detailed information on bacterial communities was obtained by sequence assignment at the species level. The heatmap in Fig. 6 shows differences in the relative abundance of the main species identified from feedlot cattle fecal samples recovered at T3 after administration of the five probiotic groups during the three experimental periods. Control group samples at T0 and T3 were included to better highlight the observed changes. In agreement with the dominance of Firmicutes and Actinobacteria, fecal samples from the control (T0) showed the highest abundance for *L. vini*, *L. casei*, *L. buchneri*, *L. coryneformis*, *L. mucosae*, *L. plantarum*, *Weissella* (*W.*) *paramesenteroides*, *W. cibaria*, *Clostridium* (*Cl*.) *tyrobutyricum*, *Cl. butyricum*, *Bifidobacterium* (*B.*) *thermacidophilum* and *B. adolescentis*; these species experienced a dramatic decrease in control samples at T3. Although probiotic supplementation did not change the richness and diversity of treated fecal samples, more ruminal fermentative bacteria (*Turicibacter sanguinis*, *Cl. dii*, *Cl. sordellii*, *Cl. tyrobutyricum*, *Cl. butyricum*, *Cl. colicanis*, *Ruminococcus* (R.) *bromii*, *R. gnavus*, *Roseburia faecis*, *Coprococcus catus*, *Eubacterium biforme*, *Blautia obeum*, *Prevotella copri*) and beneficial bacteria such as *B. adolescentis*, *B. pseudolongum* and *Fecalibacterium prausnitzii* were significantly found. These bacterial species were distributed in the heatmap from left to right in an increasing manner for the A, B, C, D and E probiotic groups; E and D exhibited the highest relative abundance. However, even at low abundance, pathogen species were also observed, and fecal samples A, B and E showed the presence of *Cl. difficile*, while a higher relative abundance of *Enterobacter*/*Echerichia*/*Shigella* was found in the D-treated sample at T3.

### LEfSe analysis

**Figure 7:**
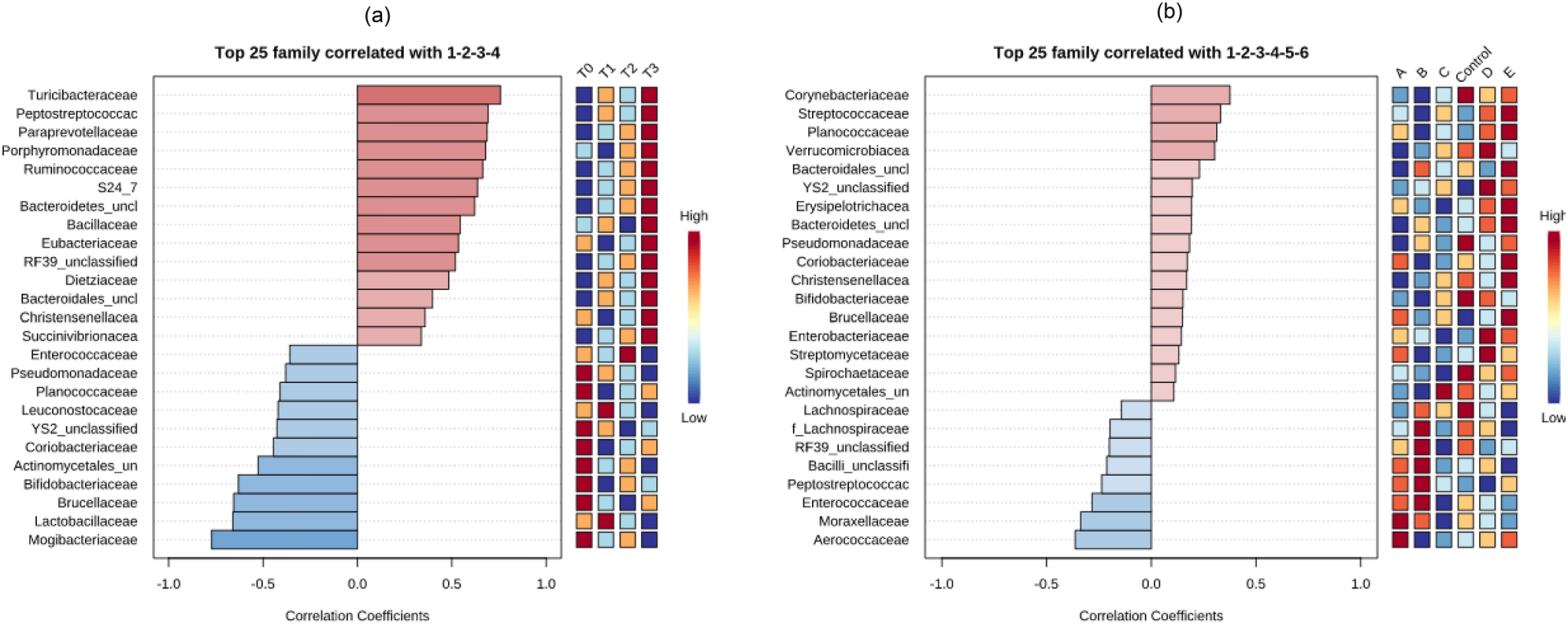
Plot from LEfSe analysis indicating enriched bacterial families associated either with positive (pink) or negative (light blue) correlations in cattle fecal samples as affected by (**a**) experimental administration periods and (**b**) probiotic groups.

Among the top 25 families, the LEfSe analysis plot (Fig. 7) for probiotic administration periods and probiotic groups showed that family relative abundance was associated with either positive or negative correlations. On this basis, 14 families were enriched from T0 to T3, showing *Turicibacteraceae*, *Peptostreptococcaceae*, P*araprevotellaceae*, *Porphyromonadaceae*, *Ruminococcaceae* and *S24-7* (recently described as *Muribaculaceae*) as the most positively affected (Fig. 7a). However, 11 families correlated negatively with the administration period, decreasing the abundances of *Mogibacteriaceae*, *Lactobacillaceae*, *Brucellaceae, Bifidobacteriaceae*, unclassified Actinomycetales and *Coriobacteriaceae*. On the other hand, probiotic groups administered at T3 showed *Streptococcaceae* and Planococcae highly enriched by group E and *Verrucomicrobiaceae* highly enriched by group D, while control samples exhibited the highest positive correlation with *Corynebacteriaceae* (Fig. 7b). Conversely, *Aerococcaceae* and *Moraxellaceae* families decreased their abundances when probiotic A was delivered, while group B reduced *Enterococcaceae* and *Peptostreptococcaceae* relative abundances. In agreement with that predicted by richness and diversity indices, family abundances were more affected by the time of probiotic administration than the probiotic group delivered to cattle.

### Potential functional annotations of feedlot cattle fecal microbiota

**Figure 8:**
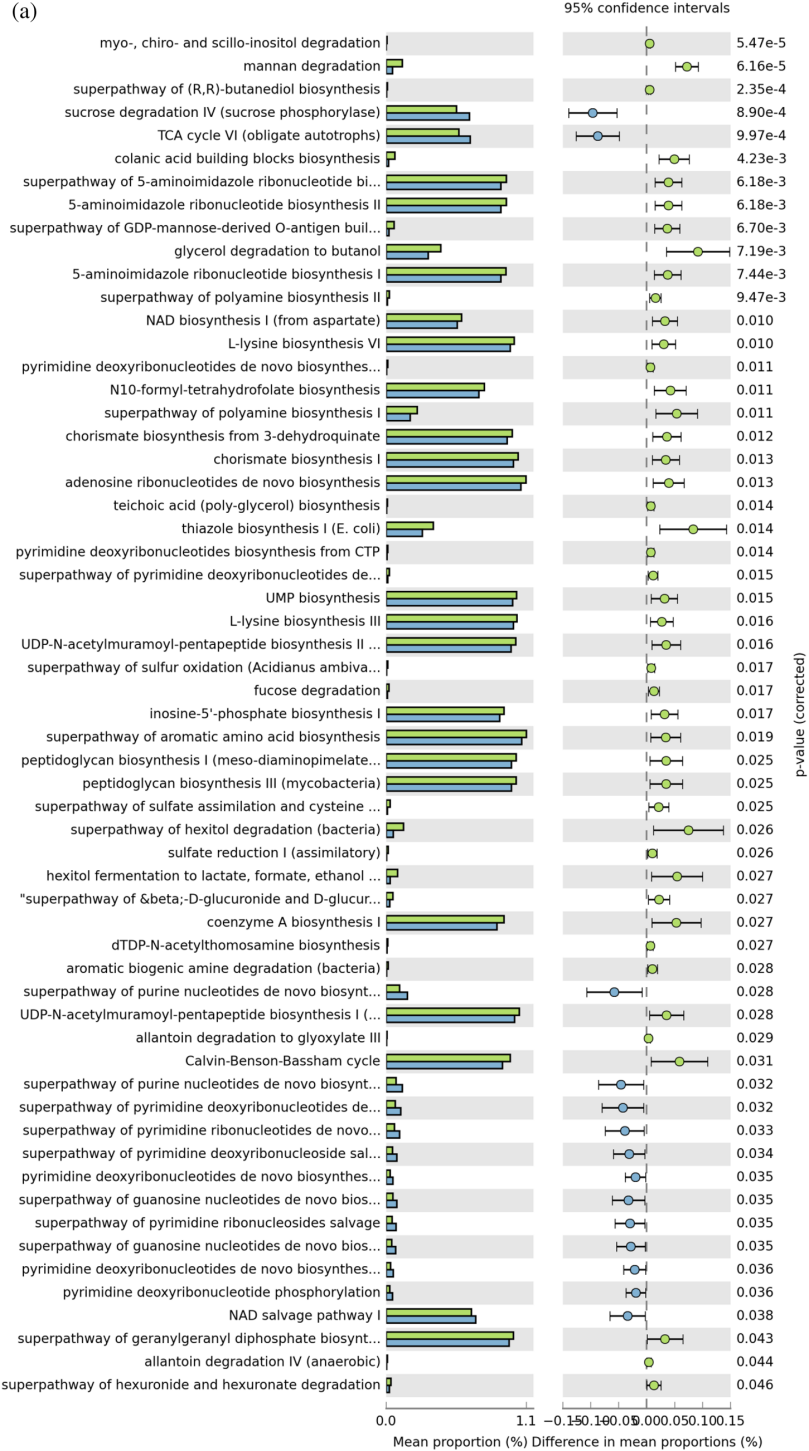

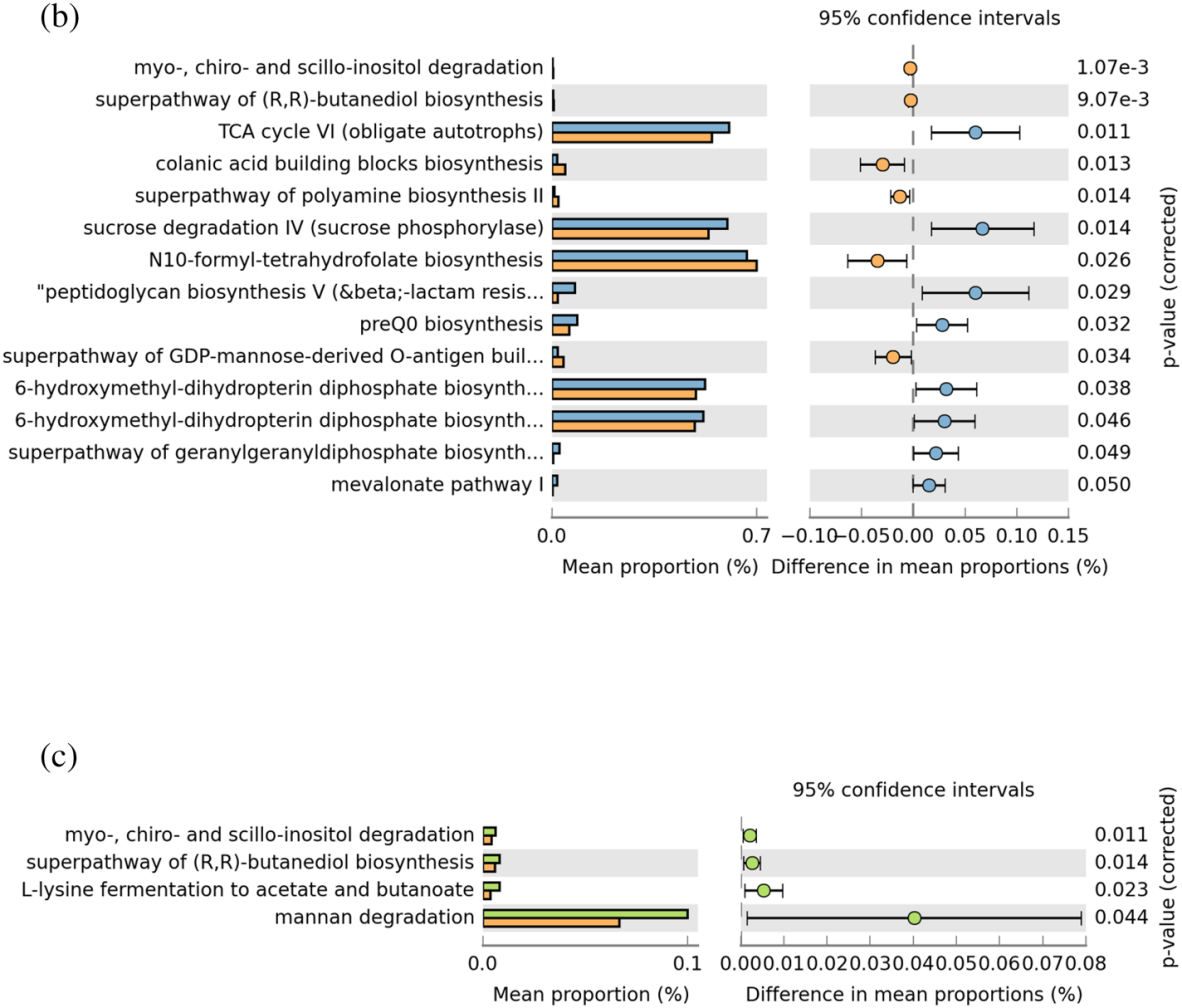
Differential PICRUSt2 predicted metacyclic pathways. Metabolic pathway comparison using STAMP between probiotic administration periods: (**a**) E-40 *vs*. E-104; (**b**) E-40 *vs*. E-163 and (**c**) E-104 *vs*. E-163.  E- 40;  E-104;  E-163. The q-values are based on Welsh's t test and corrected with Benjamini–Hochberg FDR.

The administration of probiotics did not seem to modify the structure of the intestinal microbiota detected in fecal samples; however, the different experimental periods of probiotic administration modified the abundances of the main bacterial groups identified. Therefore, to understand whether changes in taxa abundance favor positive metabolic changes contributing to improved animal health, we used PICRUSt2 for functional metabolic pathway prediction from the 16S data. The analyses included samples collected at 163 days (T3) after 40, 104 and 163 days of probiotic treatment. The pairwise comparison of microbial MetaCyc pathway abundance revealed that for all probiotic treatments, there was an enhancement of all metabolic pathways compared with the control. MetaCyc pathway abundance for each analyzed sample, including the control (no probiotic administration), is shown in Table S1. Comparison of microbial pathway abundance between the E-40 vs E-104, E-40 vs E-163 and E-104 vs E-163 experimental periods was performed to better examine the influence of the different periods of probiotic administration on the metabolic functions of the fecal microbiota. There were substantial differences (two-sided Welch's t test; p < 0.05) in 59 out of 274 pathways between the E-40 and E-104 experimental times, and most of the predicted pathways were mostly enriched in the E-40 experimental group, except for those related to nucleoside and nucleotide biosynthesis, the TCA pathway and sucrose degradation IV (Fig. 8a and Table S1). Significant differences were found comparing E-40 vs E-163 and E-104 vs E-163 (6 and 14 out of 274, respectively); pathways were mostly activated in E-40 and E-104 compared with the E-163 experimental group (Fig. 8b and c).

## Discussion

The beneficial effect of feeding probiotics to improve the health and productivity of feedlot cattle on high-grain diets has been well established^3,8,33,34^. Different lactic acid bacteria (LAB) previously selected as probiotics^31,32^ were used individually and/or as mixed cultures to supplement feedlot cattle rations in which antimicrobial treatment with the ionophore monensin was administered to animals. Recently, HTS allowed culture-independent metagenomic approaches to access the complete bacterial repertoire within fecal samples. Although the changes in feedlot cattle feces associated with different management practices and health statuses have been widely described, scarce information is available regarding changes occurring in the fecal microbiota structure and population functionality during probiotic administration. In this study, dietary supplementation with three probiotic strains, both individually and in combination, was investigated. Due to the difficulties and high economic costs involved in an in vivo study with large animals, the authors recognize the limitations in the experimental design, where a single pen, which was sampled at different times, was used to evaluate both the probiotic effect and the effect of administration time.

Fecal samples from feedlot cattle shared similar community membership and structure regardless of the probiotic group administered. Indeed, it was recently shown that shared environments have a greater impact on shaping the microbiota of individuals than host genetics^35^. The results from this study agree with the lack of significant differences in alpha diversity detected among treatments when *Saccharomyces cerevisiae* fermented product, *L. casei* + *L. plantarum*, *B. subtilis* and *E. faecium* alone or combined with *Cl. butyricum* were fed to steers, dairy cows, broilers and piglets, respectively^36–40^. In addition, the distinct clustering patterns that separated the fecal microbiota on E-40 from those on E-104 and E-163 were correlated with the significant differences in beta diversity following supplementation with *E. faecium* and *Cl. butyricum* to postweaning pigs^40^. Thus, a greater influence of administration time compared to that of probiotic groups on microbial diversity of fecal samples was observed in this study.

In line with previous reports on the fecal microbiome from meat animals, a small number of phyla accounted for the majority of the intestinal microbiota. By applying HTS, fecal samples collected from feedlot cattle administered five probiotic groups provided a detailed view of the fecal microbiome. The dominant phyla found irrespective of probiotic group administration were Firmicutes and Actinobacteria, followed by Bacteroidetes and Proteobacteria, which varied in the number of families and genera among probiotic groups. Here, probiotic treatments showed a low impact on the microbiome composition among treatments; however, dietary supplementation with probiotics has been reported to alter the gut and fecal microbiota of postweaning pigs and broiler chickens^35,38–40^. The high dominance of Firmicutes in feedlot feces coincided with those reported for feedlot beef^23,27,41–43^ and dairy cattle^28,44^. In this study, families *Clostridiaceae*, *Turibacteraceae*, *Lactobacillaceae* and *Ruminococcaceae* were predominantly represented in probiotic-administered fecal samples, in agreement with that previously described for the fecal microbiota of feedlot cattle fed high-energy diets^14,41,42^. Indeed, the relative abundance of *Clostridiaceae*/*Clostridium* can influence both positively and negatively the animal host, which is associated with the involved species. As butyric acid producers and beneficially cellulose digestion enhancers, the presence of *Cl. tyrobutyricum* and *Cl. butyricum* agrees with those reported in bovine gut and feces^45,46^. On the other hand, *Cl. difficile* found in probiotic-treated samples correlated with the high prevalence reported in beef cattle at slaughter, highlighting food contamination potential^47,48^. In accordance with abundances in the core microbiome and LEfSe analysis, a bloom of *Turicibacteraceae* from T0 to T3 in all fecal samples was found. *Turicibacter* has been detected as the dominant genus in the GIT of broilers, cattle rumen and feces^29,39,43,49^, particularly when high-grain diets were fed to ruminants^50,51^. A high abundance of *Turicibacter sanguinis* across all fecal samples was shown; however, it was higher in fecal samples after probiotic C (CRL2069) treatment at T3. A contradictory presumption as a pathogen for this genus was reported^52^; hence, the potential negative effects on cattle fed grain-rich diets health/performance need to be investigated. In coincidence with LEfSe analysis, the high initial abundance and the subsequent reduction of *Lactobacillaceae* in feedlot fecal samples agrees with the high predominance during the preweaned period of calves, which decreased over time due to weaning and milk feeding reduction^23,25,53^. However, variable increases in *Enterococcus*, *Pediococcus* and *Streptococcus* in probiotic-treated fecal samples were observed. In agreement, most LAB found in feedlot fecal samples were also recovered from bovine gut and/or feces^51,54^. Even at lower abundance, *Ruminococcaceae*, *Lachnospiraceae*, *Mogibaceriaceae* and *Leuconostoccacea*e were found in all feedlot cattle fecal samples at T3. These bacterial successions suggested that both diet and gut development drive changes in the bacterial composition during life^55^. The presence of *R. bromii* in the feces of feedlot cattle coincide with that reported for cattle fed wet-distilled grain diets, with a key role on recalcitrant starch in the gut^42^. Moreover, a beneficial in vitro interaction was observed between *R. bromii* and *R. gnavus*, with the latter being able to benefit from starch degradation products released by *R. bromii*^56^. Within *Ruminococcaceae*, the presence of *F. praunitzii* identified from probiotic-treated fecal samples was reported to correlate with health and weight gain in ruminants, exerting intestinal anti-inflammatory effects^8,53^ In addition, a variable *Lachnospiraceae* to *Christensenellaceae* ratio was detected in all fecal samples, and their presence agrees with those found in the fecal and GIT microbiomes of ruminant and nonruminant herbivorous livestock, particularly high feed efficiency steers, dairy cattle and high-grain fed lambs^28,44,57–60^. These two families play an important role in starch and fiber gut degradation and are commonly associated with animal and human health^25,58,61^. Moreover, even when the asaccharolytic *Mogibacteriaceae* family was found in all fecal samples and negatively correlated with the administration time, its function in the ruminant gut is scarcely known^57^.

As the second predominant phylum, the presence of Actinobacteria is consistent with previous reports for young mammals as healthy infants and calves^25,62^. However, these findings disagree with other reports for postweaning calves, beef and dairy cattle fecal microbiota, in which Bacteroidetes was the second dominant phylum^23,27,28,41^. The reduction of Actinobacteria in cattle fecal samples indicate that its presence is age-dependent, being higher during the first weeks of calf life^24,25,63^. Within this phylum, *Bifidobacteriaceae* and *Coriobacteriaceae* exhibited the highest relative abundance, the former being the most prevalent. The *Bifidobacterium* genus, even negatively correlated with the administration time in this study, has a pivotal role in complex carbohydrate metabolism and the maintenance of gut homeostasis; these positive features in the gut have been associated with good health of the host^64,65^. Species of *Bifidobacterium* found here were reported to be widely distributed as commensals in the gut and feces of humans and animals^66–68^. In addition, *Coriobacteriaceae* abundance agrees with the high relative abundance reported for pasture-fed dairy cows compared to grains fed diets^44,69^. This family plays important functions in the gut, such as the conversion of bile salts and phytoestrogens as well as the activation of dietary polyphenols; the production of homologs of vitamin K2 also seems to be beneficial^70^. Changes in the relative abundance of Firmicutes and Actinobacteria phyla in fecal samples reflect the gradual adaptation of calves’ GIT, first to milk and later to solid feed consumption. In the commercial feedlot, arriving young steers experienced changes in the diet from forage (silage/hay) to an increasing grain concentration diet (cracked corn, 16 to 78%; soybean expeller, 8 to 10%). Starch abundance and digestibility in grain-fed cattle compared to fiber in forage diets will induce different microbial compositions in fecal bacterial structure and phylum subpopulations responding differently^41^. Indeed, across a starch gradient, Firmicutes changed in composition and decreased in abundance, while no changes in relative abundance were shown for Actinobacteria, but their composition did^41^. In addition, a lower abundance of *Corynebacteriaceae* was also retrieved from steer feces, and *Corynebacterium* (*Co*.) *marinum*, *Co. maris* and *Co. stationis* were present in control and probiotic-treated samples at T2 and T3. This Actinobacteria genus was reported from perinatal intestinal and cow udder microbiota, indicating the presence of dysbiosis and lower feed efficiency beef cattle^62,71,72^. In this study, the Firmicutes to Actinobacteria ratio after probiotic administration decreased toward the end of the fattening cycle, and the highest richness of Actinobacteria was found for probiotic groups at T2 and T3.

In contrast, the relative abundance of the phylum Bacteroidetes as a third population exhibited an increased pattern in probiotic-administered fecal samples at T3. In coincidence with^41^, this phylum tended to increase in abundance paralleling the grain proportion in the diet. In other studies, it was reported as the second population following Firmicutes, having a high relative abundance in dairy cows, calves^38,43,44^ and high grain-fed beef cattle^42,69^. Even when families from this phylum were not part of the fecal core microbiome, *S24-7* or *Muribaculaceae* family^73^ and unclassified Bacteroidetes showed the highest richness in treated fecal samples; probiotic groups A and D had the greatest relative abundance at T3. The *Prevotella* genus detected in much lower abundance agrees with that found in feces from beef cattle fed high-grain diets, which was reported to aid the host in the digestion of complex carbohydrates such starch^41–43,74^; in particular, *Prevotella copri* identified here has been associated with cattle fed high-energy diets^60^. In coincidence with the scarce recovery of *Prevotella* in the presence of probiotics, a reduction of this genus after active dry yeast supplementation to lactating dairy cows fed a high-grain diet, was reported^75^.

Furthermore, the Proteobacteria phylum represented in this study by *Enterobacteriaceae*/*Enterobacter* showed the highest abundance for the A and D probiotic-treated samples at T3. The presence of *Gammaproteobacteria* members coincided with those reported for feces of grains fed cattle^76,77^. It is known that a Proteobacteria increase in the gut may be indicative of dysbiosis facilitating inflammation or invasion by pathogens^78^. Unsurprisingly, fecal samples after probiotic group C (*L. mucosae* CRL2069) showed the lowest abundance, coinciding with the immunobiotic ability of this probiotic strain protecting bovine host against TLR-mediated intestinal inflammatory damage, as was recently described^17^. In addition, the decreased abundance of the Tenericutes phylum at T3 in feedlot fecal samples correlates with that reported for beef and dairy cattle feces^28,29,74^. Bacteria lacking peptidoglycan cell walls and typically parasites or commensals of eukaryotic hosts are considered potential pathogens, and their function in the gut remains unclear^79^. Other phyla in low abundance included Spirochaetes and TM7 and were found in all fecal samples, while Verrucomicrobia, Chloroflexi and Cyanobacteria were not. Even with a poor physiology understanding, the TM7 phylum appears to be ubiquitous in the GIT of mammals and is reported as part of the bacterial community of lactating dairy cows and beef cattle^69,71^.

Identifying a core microbiome is the first step to predict its response to perturbations and to define a microbial community that will guide its manipulation for desired outcome achievement^80^. The presence of core taxa across fecal samples indicates that these microorganisms perform metabolic functions universal to the collective cattle fecal microbiome. In this study, although sequences were assigned to numerous taxa, only a small number of them (2 phyla, 5 orders, 11 families, and 27 genera) were detected across all cattle fecal samples. Firmicutes and Actinobacteria represented two of the ten phyla found across all fecal samples of feedlot cattle administered probiotics, while no taxa from Bacteroidetes were involved. Nevertheless, Bacteroidetes was reported as a core phylum in the gut of bovines fed high-energy diets^69^. Indeed, in the absence of probiotics, the *Prevotella* genus was consistently found as part of the core microbiome in the feces of beef cattle fed high-energy diets^41,42^. In this study, probiotic administration to feedlot cattle seemed to stimulate the presence of “health-associated” bacterial species such as those from *Ruminococcaceae*, *Lachnospiraceae* and *Bifidobacteriaceae* identified among the core microbiome. These results are in accordance with the modulatory effect of feed additives such as phytobiotics and live/autolyzed yeast used in cows fed high-grain diets, which increased the abundance of gram-positive bacteria and decreased that of gram-negative bacteria^9^. Moreover, oral administration of probiotics (*L. plantarum*) to dairy calves was found to affect the rumen bacterial community with a decrease in the numbers of cellulolytic bacteria^81^. As an important component of the diet, it is worth noting that the presence of ionophore monensin could also be involved in fecal microbiome changes, since it has been shown to alter digestive microbiota in cattle fed a forage diet of members of the phylum Bacteroidetes^82^. However, it seems unlikely that the presence or absence of an ionophore is the sole explanation for shifts in the population compositions of Firmicutes and Bacteroidetes. Other factors, such as cattle breeding, environment, feeding practices and management^24,25,41,42,69,83^, may also account for the succession in phyla abundance found in this study.

It is known that the cattle GIT microbiome fulfills many physiological functions that are lacking in the host; thus, they are considered essential to cattle life. To determine the potential functions of the microbiota identified from fecal samples, PICRUSt2 was used to infer putative abilities by prediction of functional genes that are typically associated with different taxa. The main metabolic pathways found in this study are consistent with the role of these functions in microbial life^84^. Abundances affected by the different experimental periods of probiotic administration were compared, since a higher impact on fecal microbiome structure was before determined. Pairwise comparisons among administration periods revealed significant differences in bacterial functions; the E-40 period exhibited the highest number of metabolic pathways. Carbohydrate degradation (mannan, fucose, glycerol, hexitol) pathways agree with the high forage rich in cellulose and hemicellulose (> 60%) fed to feedlot cattle; the early high presence of Firmicutes/Clostridiales in the fecal microbiome would account for these activities. Similar results were reported for the functional capacity of the gut/fecal microbiota of ruminants, swine and broilers^28,84–86^. In addition, biosynthetic and derivative pathways related to amino acids and protein metabolisms detected were higher in the E-40 experimental period. As was reported^61,71^, *Lachnospiraceae*, *Ruminococcaceae* and mostly *Clostridiaceae* are commensal with a high role in the digestion of carbohydrates and proteins, which agrees with their richness as microbiome core members. Species from these families were associated with better feed efficiency or weight average daily gain, as fermenters of a range of complex plant-derived polysaccharides with production of short-chain fatty acids, particularly butyrate, that will be used as energy source^71,86^. As a result, it may be suggested that 40 days of probiotic administration to feedlot cattle is optimal for the activation of beneficial metabolic pathways by stimulating the growth of health-associated bacteria, contrary to that observed at E-104. These results could be explained by the large decrease in richness and in diversity evidenced by the Chao1 and Shannon index, respectively, observed after 104 days compared to 40 and 163 days of probiotic administration. In coincidence, 40 days of administration of probiotic group A was also shown to be enough for a positive modulation of the microbiome according to the similar relative abundance at the genus level obtained from 40 to 163 days.

## Materials and methods

### Probiotic bacteria, culture conditions and inoculum preparation

*Lactobacillus (L.) acidophilus* CRL2074, *Limosilactobacillus* (*L*.) *fermentum* CRL2085 and *Limosilactobacillus (L.) mucosae* CRL2069 (formally named *Lactobacillus fermentum* and *Lactobacillus mucosae*, respectively) previously isolated from feedlot cattle^31^ were used as probiotics. Inoculi of strains were prepared by transferring glycerol stock culture to MRS broth (Biokar, France) and subcultured twice in the same media at 37 °C for 18 h. Inoculi from each LAB probiotic strain were used for multiplication at a CERELA (CONICET) pilot plant (data not shown). The obtained concentrated cell mass for each probiotic strain was distributed in plastic containers of 100 g each, with a cell concentration between 10^10^ and 10^11^ cfu/g and stored at -20 °C until use. Cell viability before administration was determined.

### Animal’s experimental design

A total of 126 Brangus and Braford (crossbreed Brahman/Angus and Brahman/Hereford, respectively) steers were eligible for inclusion in this study. Cattle belonged to a commercial feedlot located in the Northern Province of Santiago del Estero (Argentina). Upon arrival, control of animal health (vaccination against infectious organisms, respiratory diseases, and parasites) was carried out according to the livestock preventive sanitary plan developed by the veterinary staff of the feedlot. After the control period (day 15), animals with an initial average body weight of 150–170 kg were randomly allocated to six separate pens, containing n = 21 animals each. During the fattening cycle, in addition to urea (0.5%) and minerals/vitamins (1.7–2.0%), a high-energy diet consisting of three rations with different compositions containing decreasing sorghum silage (63 to 17%) and soy expeller (9.5 to 3%) and increasing corn cracked kernel (16.5 to 77.8%) amounts was delivered^31^. As a routine, all cattle received ionophore monensin, 14 mg/kg of total mixed ration (Rumensin 200) treatment, and feed rations were supplied twice a day. One-half of the allowed daily rations at each feeding and ad libitum access to water were provided. Administration of probiotics to feedlot cattle was carried out three days per week by suspending the content of one frozen concentrated cell pot (100 g) from each probiotic strain in a two-liter spray bottle, which was top-dressed on the feed ration mix to obtain a final probiotic concentration between 10^7^–10^8^ CFU/animal/day. The adopted probiotic administration scheme was due to operative reasons. In the commercial feedlot (15,000 animals) total mixed ration was delivery daily using a large-scale system; in this experiment a different feeding management was required, three days/week and a reduced number of animals into different pens (each probiotic assayed) were used. Prior to administration, ear tags (numbered and different colors for each probiotic group) were used for identification of each animal. The probiotic groups were (A) *L. acidophilus* CRL2074, (B) *L. fermentum* CRL2085, (C) *L. mucosae* CRL2069, (D) *L. fermentum* CRL2085 + *L. mucosae* CRL2069 and (E) *L. acidophilus* CRL2074 + *L. fermentum* CRL2085 + *L. mucosae* CRL2069; a control group (n = 21) was also included. A convenience-experimental design and sampling scheme is shown in Fig. 1 (to better understand the experimental design, a more detailed experimental protocol is shown in Fig. S1). To analyze the effect of each probiotic group, twenty-one (n = 21) bovines for each probiotic group were involved, and three experimental periods were evaluated as follows: E-40, animals (n = 7) received probiotics for up to 40 days and then removed (to separate pens); E-104, animals (n = 7) received probiotics for up to 104 days and then removed; and E-163, animals (n = 7) administered probiotics for 163 days. Fecal samples were obtained at 0 (T0), 40 (T1), 104 (T2) and 163 (T3) days, corresponding to 15, 55, 119 and 179 days of animal lot feeding, respectively (Fig. 1). Animals in experiments E-40 and E-104 were also sampled at time T3. A control group (n = 21) was included in a separate pen and analyzed from T0 to T3. Collected rectal fecal samples were stored under refrigeration (5 ºC) and sterile transported to the laboratory for further use. For metagenomic analysis, fecal samples from seven animals were pooled at each experimental time for each probiotic group. The higher cost associated with shotgun metagenome sequencing restricted the use of a larger number of samples.

### HTS analyses of bovine fecal samples

Fecal samples from the seven steers at each sampling time and for each assayed probiotic group were pooled, thus giving a total of 34 samples that were subjected to high-throughput sequencing (HTS). Total bacterial DNA was extracted using the FastDNA SPIN kit and Fast-Prep Instrument (Qbiogene, Inc., CA) according to the manufacturer’s instructions, examined on agarose gel and quantified using the Quant-iT HS ds-DNA assay kit (Invitrogen, Paisley, UK) in combination with the QuBit fluorometer. The bacterial V3-V4 16S rRNA region was amplified with the primer pairs 343F (5′-TACGGRAGGCAGCAG-3′) and 802R (5′-TACNVGGGTWTCTAATCC-3′) using Phusion Flash High-Fidelity MasterMix (Thermo Fisher Scientific, Inc. Waltham, MA, USA). A two-step nested PCR was applied, and the conditions used for reaction mix and amplification experiments were those previously described^87^. The PCR products of the second step for all samples were multiplexed in a single pool in equimolar amounts based on the QuBit quantification data. The PCR product pool was then purified using the solid phase reverse immobilization method of the Agencourt AMPure XP kit (Beckman Coulter, Italy) and sequenced at Fasteris SA (Geneva, Switzerland). The TruSeq rDNA sample preparation kit (Illumina Inc., San Diego, CA) was used for amplicon library preparation, while the sequencing reaction was performed with a MiSeq Illumina instrument (Illumina Inc., San Diego, CA) with V3 chemistry, generating 300 bp paired end reads.

### Bioinformatics and statistical analysis

HTS data filtering, multiplexing, and preparation for concomitant statistical analyses were carried out as previously described^87^. In summary, paired reads were assembled to reconstruct the full V3-V4 amplicons with the “pandaseq” script^88^, allowing a maximum of 2 mismatches and at least 30 bp of overlap between the read pairs. Sample demultiplexing was then carried out with the Fastx toolkit (http://hannonlab.cshl.edu/fastx_toolkit/). Mothur v.1.32.1^89^ was applied to remove sequences with large homopolymers (≥ 10), sequences that did not align within the targeted V3-V4 region, chimeric sequences^90^ and sequences that were not classified as bacterial after alignment against the Mothur version of the RDP training data set. For taxonomy-based analyses, the QIIME formatted Greengenes v.13.8 database was used. Taxonomies were adapted to QIIME taxonomy standards uniforming to the 7 main taxa ranks (superkingdom, phylum, class, order, family, genus, and species). The operational taxonomic units (OTUs) were identified against reference databases (Greengenes v.13.8 database) using NCBI-Blast v2.2.27 (Basic Local Alignment Search Tool of National Center for Biotechnology Information online website). After counting the abundance of each OTU, a final OTU-table output file was created using custom scripts. Community alpha diversity was estimated using Chao1 (species number abundance in a community) and Shannon (used to quantify specific biodiversity) indices. The beta diversity was evaluated with the Bray–Curtis similarity index, which was used for all nonparametric data. Statistical differences in bacterial community composition between samples from the probiotic groups were calculated using analysis of variance (PERMANOVA) with 9999 permutations; principal coordinate analysis (PCoA) of relative abundance at the family level was performed^93^. For all statistical comparisons, differences with *p* < 0.05 were considered significant. These tests were performed using the QIIME package, version 1.5.0 in the pipeline Microbiome Analyst (http://microbiomeanalyst.ca/faces/home.xhtml). The relative abundances of OTUs between groups were compared with the Kruskal–Wallis test.

Linear discriminant analysis effect size (LEfSe)^91^ was used to identify bacterial taxa that were positively and negatively enriched in feedlot cattle feces after probiotic administration and during different periods of administration. Phylogenetic Investigation of Communities by Reconstruction of Unobserved States (PICRUSt2) was used for predicting the functional profiling of microbial communities on the 16S rDNA sequences^92^. Functional profiling was built based on the MetaCyc Metabolic Pathway Database^93^. We used the STAMP v2.1.3 software package to analyze the metabolic potential of the microbial communities. 

### Ethics approval and consent to participate

The in vivo tests were carried out according to the recommendations of the Argentine Association for the Science and Technology of Laboratory Animals, which is based on the National Institutes of Health Guide for the Care and Use of Laboratory Animals and the Federation of European Associations of Laboratory Animal Sciences (NIH Publications No. 8023, 1978). The protocol applied in the experimental trials was evaluated and approved by the CERELA ethics committee and CICUAL (Institutional Committee for the care and use of laboratory animals CIUCUAL-UNT, Argentina, Resolution 025/2017 and 0920/2017). All animal protocols used in this study were in compliance with the ARRIVE guidelines.

## Conclusions

To our knowledge, this is the first investigation on the effect of lactobacilli probiotic administration to feedlot cattle fed a forage decreasing and grains increasing diet during a fattening cycle. Fecal samples after probiotic treatments showed no significant differences in their bacterial diversity, although significant differences were identified when examining the influence of administration periods. However, a microbiome core set of bacteria from the Firmicutes and Actinobacteria phyla was shared across all cattle fecal samples administered probiotics, implying that these microorganisms were involved in performing fundamental metabolic functions essential to the collective cattle microbiome. The cattle fecal microbiome after probiotic administration increased the abundance of Firmicutes, enhanced the presence of Actinobacteria and reduced the abundance of Bacteroidetes. In addition, probiotic delivery during the first 40 days seems to contribute to the metabolic pathways needed to enhance the presence of health- and growth-associated bacteria in feedlot cattle. The benefit of lactic acid bacteria as probiotics in feedlot cattle was evident, and even though these results are promising for antibiotic substitution, an in-depth analysis of the mechanisms underlying the probiotic effect in the cattle gut and a comparison of probiotic administration in the presence and absence of the antibiotic monensin remain to be investigated.

## Supplementary Information

Supplementary Information 1.

Supplementary Information 2.

Supplementary Information 3.

Supplementary Information 4.

Supplementary Information 5.

## Data Availability

The data generated or analyzed during the current study are available from the corresponding author by reasonable request.

## Abbreviations

HTS: High-throughout sequencing
16S rDNA: 16S ribosomal deoxyribonucleic acid
GIT: Gastrointestinal tract
LAB: Lactic acid bacteria
TLR: Toll-like receptors
EHEC: Enterohemorrhagic
DNA: Deoxyribonucleic acid
dNTPs: Deoxynucleotide triphosphates
OTU: Operational taxonomic unit
PCoA: Principal coordinate analysis
PCR: Polymerase chain reaction
RDP: Ribosomal data project
LEfSe: Linear discriminant analysis effect size
QIIME: Quantitative insights into microbial ecology
PERMANOVA: Permutational multivariate analysis of variance
PICRUSt: Phylogenetic investigation of communities by reconstruction of unobserved states
STAMP: Statistical analysis of metagenomic profiles
