# Supplementary material for: Administration of probiotic lactic acid bacteria to modulate fecal microbiome in feedlot cattle
Source: Sci Rep. 2022 Jul 28;12:12957. doi: 10.1038/s41598-022-16786-z (PMC9334624; doi:10.1038/s41598-022-16786-z)
Supplement: Supplementary file 1 — Supplementary Information 1. [file 41598_2022_16786_MOESM1_ESM.pdf]

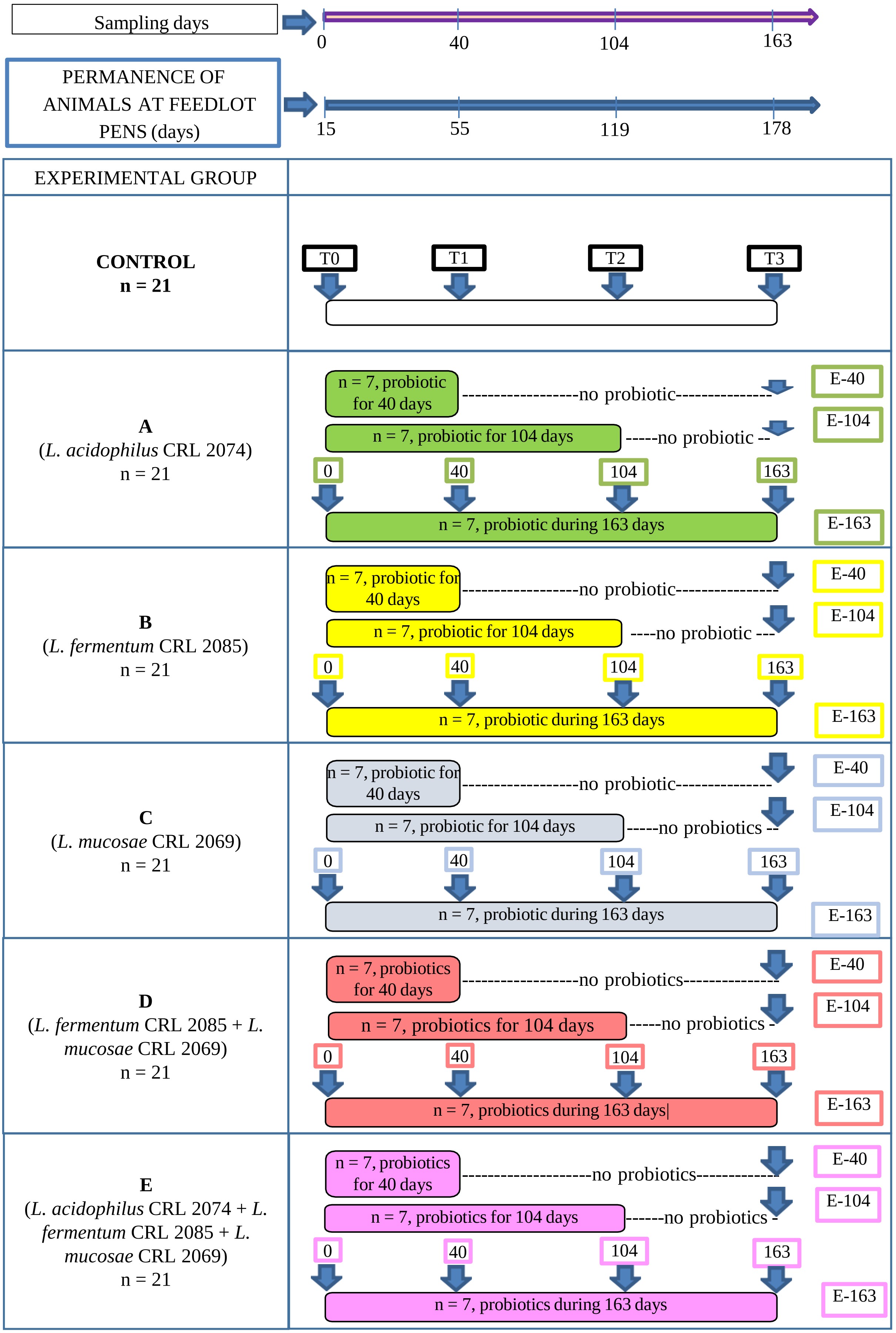
Sampling days
0
40
104
163
PERMANENCE OF ANIMALS AT FEEDLOT PENS (days)
15
55
119
178
EXPERIMENTAL GROUP
CONTROL
n = 21
T0
T1
T2
T3
A
(L. acidophilus CRL 2074)
n = 21
n = 7, probiotic for 40 days
-----------------no probiotic---------------
n = 7, probiotic for 104 days
-----no probiotic --
0
40
104
163
n = 7, probiotic during 163 days
E-40
E-104
E-163
B
(L. fermentum CRL 2085)
n = 21
n = 7, probiotic for 40 days
-----------------no probiotic----------------
n = 7, probiotic for 104 days
----no probiotic ---
0
40
104
163
n = 7, probiotic during 163 days
E-40
E-104
E-163
C
(L. mucosae CRL 2069)
n = 21
n = 7, probiotic for 40 days
-----------------no probiotic---------------
n = 7, probiotic for 104 days
-----no probiotics --
0
40
104
163
n = 7, probiotic during 163 days
E-40
E-104
E-163
D
(L. fermentum CRL 2085 + L. mucosae CRL 2069)
n = 21
n = 7, probiotics for 40 days
-----------------no probiotics---------------
n = 7, probiotics for 104 days
-----no probiotics -
0
40
104
163
n = 7, probiotics during 163 days|
E-40
E-104
E-163
E
(L. acidophilus CRL 2074 + L. fermentum CRL 2085 + L. mucosae CRL 2069)
n = 21
n = 7, probiotics for 40 days
-------------------no probiotics-------------
n = 7, probiotics for 104 days
------no probiotics -
0
40
104
163
n = 7, probiotics during 163 days
E-40
E-104
E-163

Fig. S1

Fig. S1 Detailed experimental protocol and sampling scheme: animals (n = 7) received probiotics up to 40 days and then removed (to separate pen) experimental period E-40; animals (n = 7) received probiotics up to 104 days and then removed, E-104 and, animals (n = 7) administered probiotics for 163 days E-163. This scheme was replicated for the following probiotic groups: A: *Lactobacillus* (*L.*) *acidophilus* CRL 2074; B: *Limosilactobacillus* (*L*) *fermentum* CRL 2085; C: *Limosilactobacillus* (*L*) *mucosae* CRL 2069; D: CRL 2085 + CRL 2069; E: CRL 2074 + CRL 2085 + CRL 2069; Control group (n = 21) included in a separate pen and analyzed from T0 to T3.
