# Supplementary material for: Administration of probiotic lactic acid bacteria to modulate fecal microbiome in feedlot cattle
Source: Sci Rep. 2022 Jul 28;12:12957. doi: 10.1038/s41598-022-16786-z (PMC9334624; doi:10.1038/s41598-022-16786-z)
Supplement: Supplementary file 2 — Supplementary Information 2. [file 41598_2022_16786_MOESM2_ESM.pdf]

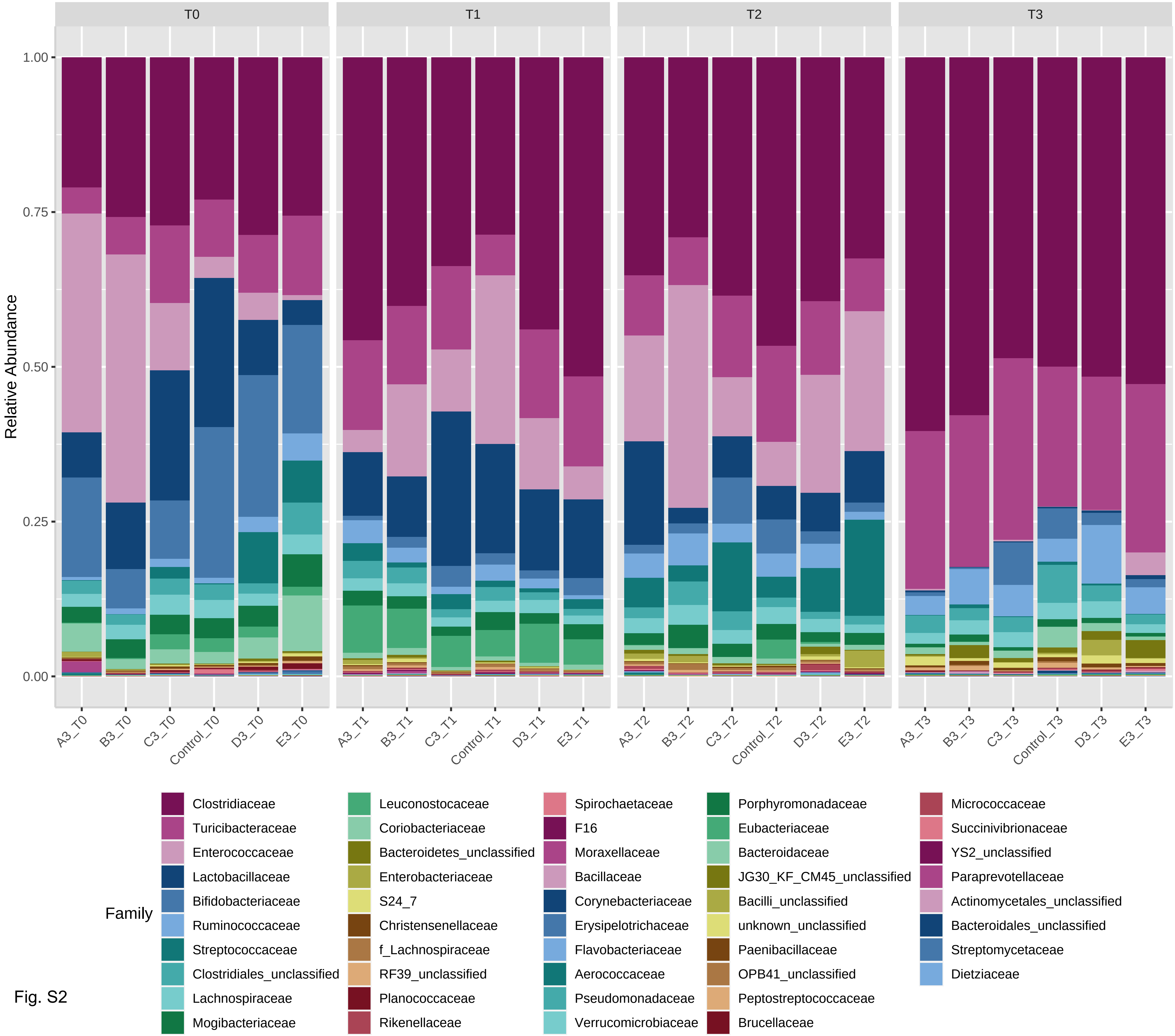
T0
T1
T2
T3
Relative Abundance
1.00
0.75
0.50
0.25
0.00
A3_T0
B3_T0
C3_T0
Control_T0
D3_T0
E3_T0
A3_T1
B3_T1
C3_T1
Control_T1
D3_T1
E3_T1
A3_T2
B3_T2
C3_T2
Control_T2
D3_T2
E3_T2
A3_T3
B3_T3
C3_T3
Control_T3
D3_T3
E3_T3
Family
Clostridiaceae
Turicibacteraceae
Enterococcaceae
Lactobacillaceae
Bifidobacteriaceae
Ruminococcaceae
Streptococcaceae
Clostridiales_unclassified
Lachnospiraceae
Mogibacteriaceae
Leuconostocaceae
Coriobacteriaceae
Bacteroidetes_unclassified
Enterobacteriaceae
S24_7
Christensenellaceae
f_Lachnospiraceae
RF39_unclassified
Planococcaceae
Rikenellaceae
Spirochaetaceae
F16
Moraxellaceae
Bacillaceae
Corynebacteriaceae
Erysipelotrichaceae
Flavobacteriaceae
Aerococcaceae
Pseudomonadaceae
Verrucomicrobiaceae
Porphyromonadaceae
Eubacteriaceae
Bacteroidaceae
JG30_KF_CM45_unclassified
Bacilli_unclassified
unknown_unclassified
Paenibacillaceae
OPB41_unclassified
Peptostreptococcaceae
Brucellaceae
Micrococcaceae
Succinivibrionaceae
YS2_unclassified
Paraprevotellaceae
Actinomycetales_unclassified
Bacteroidales_unclassified
Streptomycetaceae
Dietziaceae

Fig. S2

Fig. S2 Relative abundance data at the family level in probiotics (A: *Lactobacillus acidophilus* CRL 2074*;* B: *Limosilactobacillus fermentum* CRL 2085*;* C**:** *Limosilactobacillus mucosae* CRL 2069*;* D**:** CRL 2085 + CRL 2069*;* E**:** CRL 2074 + CRL 2085 + CRL 2069)-treated and untreated (control) fecal samples of feedlot cattle at different experimental periods (T0: 0 day; T1: 40 days; T2: 104 days; T3: 163 days). Each color represents a family.
