# Supplementary material for: Administration of probiotic lactic acid bacteria to modulate fecal microbiome in feedlot cattle
Source: Sci Rep. 2022 Jul 28;12:12957. doi: 10.1038/s41598-022-16786-z (PMC9334624; doi:10.1038/s41598-022-16786-z)
Supplement: Supplementary file 3 — Supplementary Information 3. [file 41598_2022_16786_MOESM3_ESM.pdf]

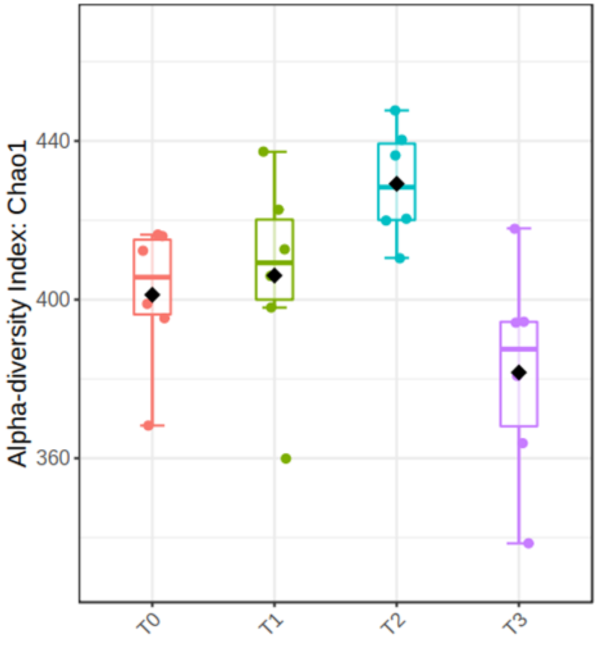
Alpha-diversity Index: Chao1
440
400
360
T0
T1
T2
T3

(a)

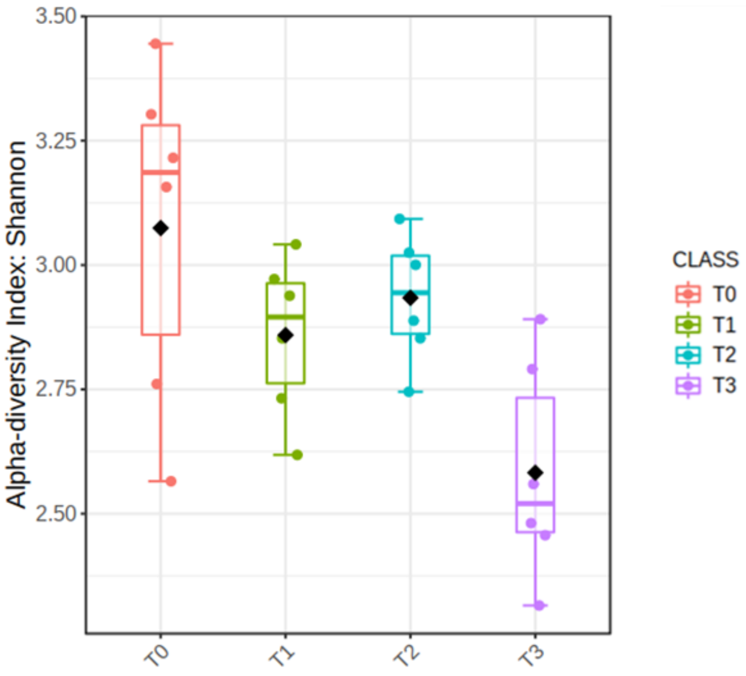
Alpha-diversity Index: Shannon
3.50
3.25
3.00
2.75
2.50
T0
T1
T2
T3
CLASS
T0
T1
T2
T3

(b)

Fig. S3

Fig. S3 Box plots show Chao1 richness (a) and Shannon (b) diversity profiles considering the sampling time during 163 days of probiotic administration. The line inside the box represents the median, while the whiskers represent the lowest and highest values within the 1.5 interquartile range (IQR). Outliers and individual sample values are shown as dots. Statistical testing p value = 0.024944; [Kruskal–Wallis] statistic: 9.3533 for Chao1 and p value = 0.030836; [Kruskal–Wallis] statistic: 8.8867 for Shannon.
