# Supplementary material for: Administration of probiotic lactic acid bacteria to modulate fecal microbiome in feedlot cattle
Source: Sci Rep. 2022 Jul 28;12:12957. doi: 10.1038/s41598-022-16786-z (PMC9334624; doi:10.1038/s41598-022-16786-z)
Supplement: Supplementary file 4 — Supplementary Information 4. [file 41598_2022_16786_MOESM4_ESM.pdf]

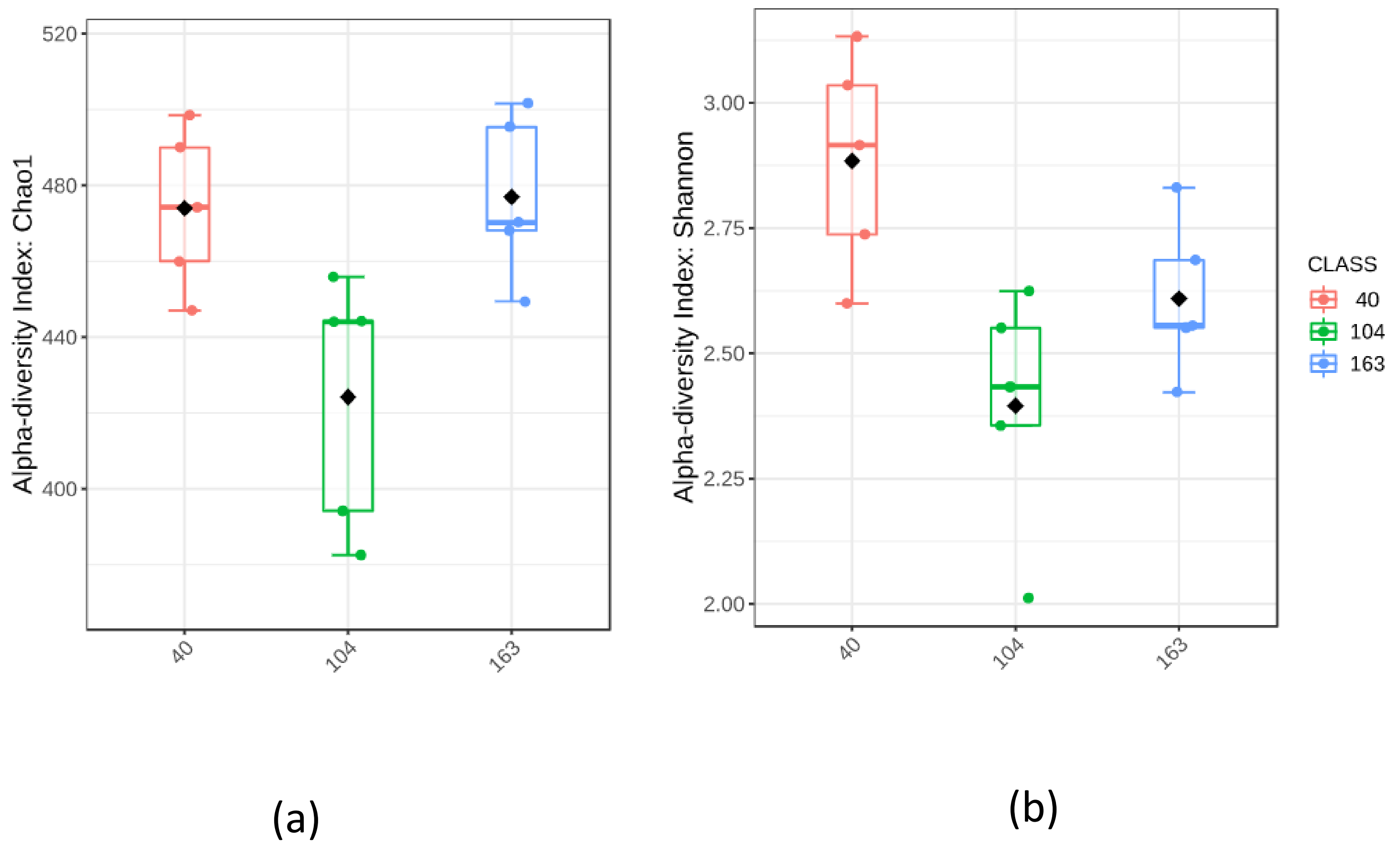

Alpha-diversity Index: Chao1
520
480
440
400
40
104
163
Alpha-diversity Index: Shannon
3.00
2.75
2.50
2.25
2.00
40
104
163
CLASS
40
104
163
(a)
(b)

Fig. S4

Fig. S4 Box plots show Chao1 richness (a) and Shannon (b) diversity profiles comparing the sampling at T3 (163 days) after three different periods of probiotic administration (E-40, E-104, E-163). The line inside the box represents the median, while the whiskers represent the lowest and highest values within the 1.5 interquartile range (IQR). Outliers and individual sample values are shown as dots. Statistical testing p value = 0.0185; [Kruskal–Wallis] statistic: 7.98 for Chao1 and p value = 0.017774; [Kruskal–Wallis] statistic: 8.06 for Shannon.
